# Supplementary material for: Gum Hydrocolloids Reinforced Silver Nanoparticle Sponge for Catalytic Degradation of Water Pollutants
Source: Polymers (Basel). 2022 Jul 31;14(15):3120. doi: 10.3390/polym14153120 (PMC9370489; doi:10.3390/polym14153120)
Supplement: Supplementary file 1 [file polymers-14-03120-s001.zip › polymers-1800320-supplementary.pdf]

Gum hydrocolloids reinforced silver nanoparticle sponge for catalytic degradation of water pollutants

Rohith K. Ramakrishnan[1], Daniele Silvestri[1], Nechikkottil S. Sumitha[2], Nhung H. A. Nguyen[1], Karel Havlíček[1], Dariusz Łukowiec[3], Stanisław Wacławek[1], Miroslav Černík[1], Diwakar Tiwari[4], Vinod V.T. Padil [1]*, Rajender S. Varma[5]*

[1] Institute for Nanomaterials, Advanced Technologies and Innovation (C×I), Technical University of Liberec (TUL), Studentská 1402/2, Liberec 1, Czech Republic, 461 17; Telephone: +420 723372911

[2] Department of Polymer Science and Rubber Technology, Cochin University of Science and Technology, Kochi-682 022, Kerala, India

[3] Materials Research Laboratory, Faculty of Mechanical Engineering, Silesian University of Technology, Konarskiego 18 a St., 44-100 Gliwice, Poland.

[4] Department of Chemistry, Mizoram University (A Central University) Aizawal- 796004, Mizoram, India

[5] Regional Centre of Advanced Technologies and Materials, Czech Advanced Technology and Research Institute, Palacký University in Olomouc, Šlechtitelů 27, 783 71 Olomouc, Czech Republic

* Corresponding Authors' Email; vinod.padil@tul.cz (Dr. Vinod V. T. Padil); Varma.Rajender@epa.gov (Prof. R. S. Varma)

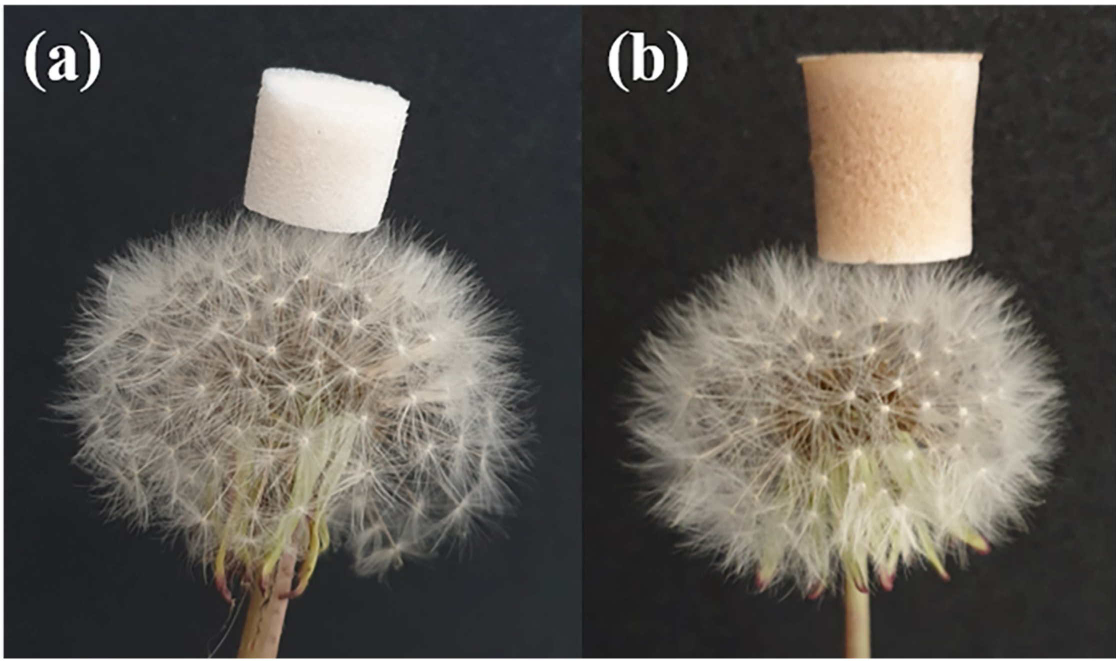

Figure S1. (a) and (b) Digital photograph of KS and Ag@KS sponge respectively on a dandelion flower

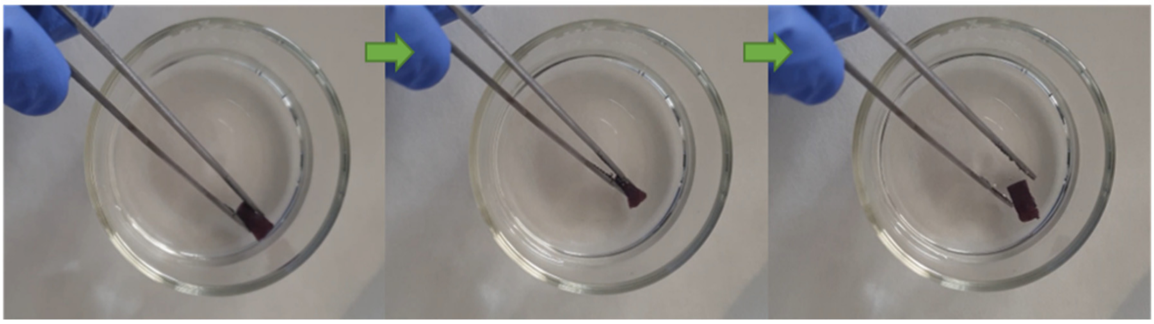

Figure S2. Photographs taken when the hydrated Ag@KS sponge was fully recovered after compressive squeezing.

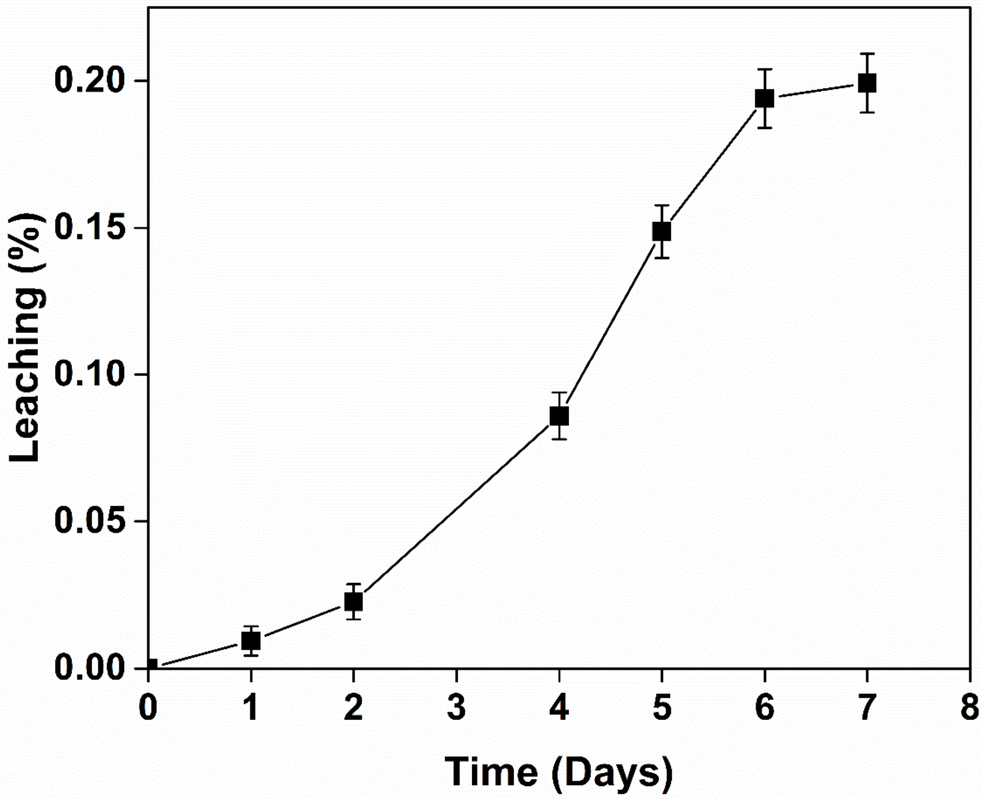

Figure S3. The leaching percentage of AgNPs from Ag@KS sponge, calculated using ICP-MS analysis for 7 days of immersion in water.

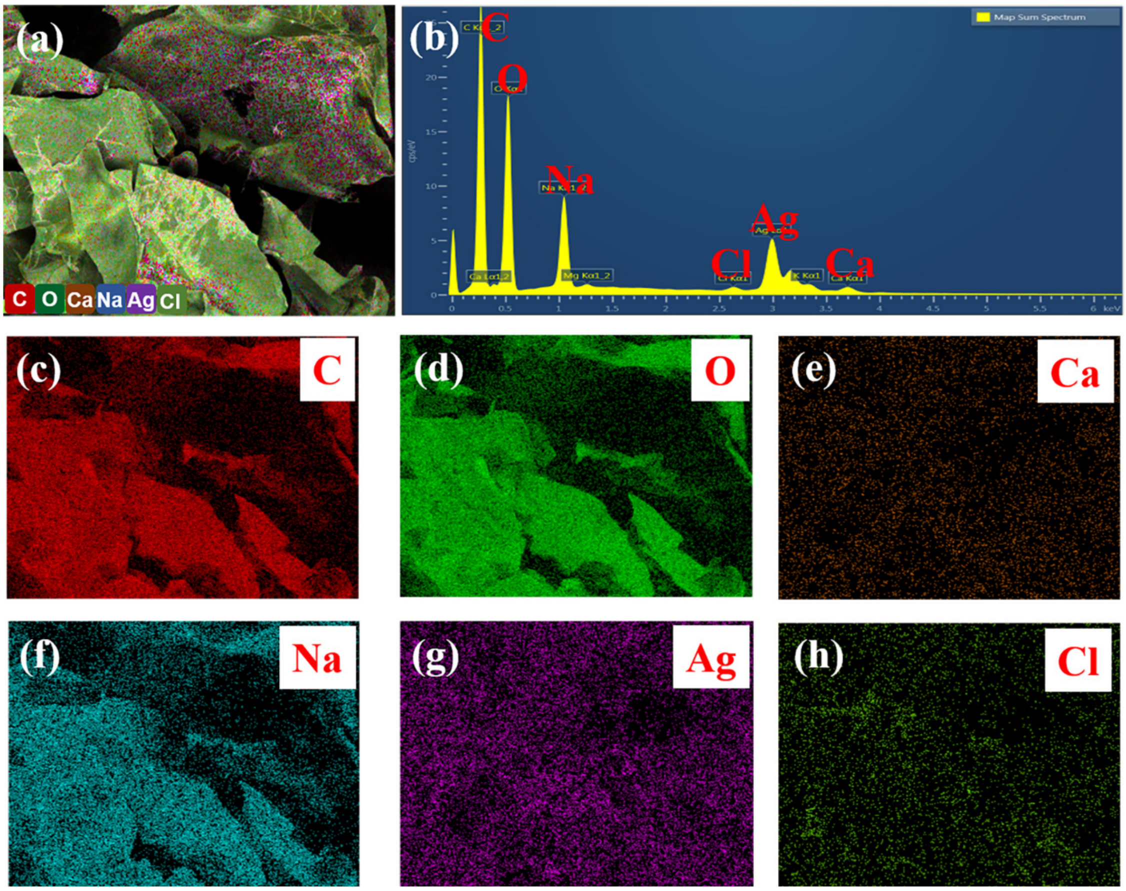

Figure S4. (a) SEM image and (b) Energy-dispersive X-ray spectra of Ag@KS sponge. Elemental distribution of (c) C, (d) O, (e) Ca, (f) Na, (g) Ag, and (h) Cl, respectively.

Table S1. The percentage of AgNPs retained in the sponge at different precursor concentrations, estimated using ICP-MS analysis.

SL. No	Concentration of $AgNO_3$ (mM)	Total polymer concentration (%)	Amount of AgNPs present in the sponge (%)
1	4	1.5	97 ± 2
2	6	1.5	96 ± 2
3	8	1.5	94 ± 1
4	10	1.5	90 ± 2

Table S2. Comparison of the catalytic activity of different catalysts for MB catalytic degradation.

**Catalyst name**	**Catalyst amount (mg)**	**Rate constant (min^{-1})**	**k_m (min^{-1} g^{-1})**	**Ref.**
Copper-agarose-sponge	20	0.5781	29	[1]
Nickel-agarose-sponge	20	0.2733	14	[1]
Iron-graphene sponge	10	0.6	60	[2]
3D-graphene/ gold nanoparticles	30	0.0125	0.4	[3]
Silver-magnetic iron oxide	10	0.34	34	[4]
Ag@KS	3.1	0.116	37	This work

Table S3. Comparison of the catalytic activity of different catalysts for 4-NP catalytic degradation.

Catalyst name	Catalyst amount (mg)	Rate constant (min^{-1})	k_m (min^{-1} g^{-1})	Ref.
Copper-agarose-sponge	20	0.3224	16	[1]
Nickel-agarose-sponge	20	0.1577	8	[1]
Copper sponge	10	0.104	10	[5]
Nickel sponge	10	0.009	1	[5]
AgNPs/graphene-Loofah sponge	76	1.893	25	[6]
Gold NPs/graphene oxide/Tannic acid	18	0.188	10	[7]
Ag@GKS	1	0.044	44	This work

Supplementary references

[1] T. Kamal, A.M. Asiri, N. Ali, Catalytic reduction of 4-nitrophenol and methylene blue pollutants in water by copper and nickel nanoparticles decorated polymer sponges, Spectrochim. Acta Part A Mol. Biomol. Spectrosc. 261 (2021) 120019. https://doi.org/10.1016/J.SAA.2021.120019.

[2] A. Naderian, S. Mohamadi, Synthesizing of Fe decorated graphene sponge for environmental applications, J. Dispers. Sci. Technol. 41 (2020) 461–469. https://doi.org/10.1080/01932691.2019.1584113.

[3] J. Xie, X. Yang, X. Xu, Wet chemical method for synthesizing 3D graphene/gold nanocomposite: catalytic reduction of methylene blue, Phys. E Low-Dimensional Syst. Nanostructures. 88 (2017) 201–205. https://doi.org/10.1016/J.PHYSE.2016.11.016.

[4] M. Zhu, C. Wang, D. Meng, G. Diao, In situ synthesis of silver nanostructures on

magnetic Fe3O4@C core–shell nanocomposites and their application in catalytic reduction reactions, J. Mater. Chem. A. 1 (2013) 2118–2125. https://doi.org/10.1039/C2TA00669C.

[5] M.T. Islam, J. Rosales, R. Saenz-Arana, R. Arrieta, H. Kim, K.A. Sultana, Y. Lin, D. Villagran, J.C. Noveron, Synthesis of high surface area transition metal sponges and their catalytic properties, New J. Chem. 43 (2019) 10045–10055. https://doi.org/10.1039/C9NJ02096A.

[6] Y.Y. Liu, Y.H. Zhao, Y. Zhou, X.L. Guo, Z.T. Chen, W.J. Zhang, Y. Zhang, J. Chen, Z.M. Wang, L.T. Sun, T. Zhang, High-efficient catalytic reduction of 4-nitrophenol based on reusable Ag nanoparticles/graphene-loading loofah sponge hybrid, Nanotechnology. 29 (2018) 315702. https://doi.org/10.1088/1361-6528/aac3e8.

[7] Y. Zhang, S. Liu, W. Lu, L. Wang, J. Tian, X. Sun, In situ green synthesis of Au nanostructures on graphene oxide and their application for catalytic reduction of 4-nitrophenol, Catal. Sci. Technol. 1 (2011) 1142–1144. https://doi.org/10.1039/C1CY00205H.
